# Molecular Epidemiology and Evolution of Swine Influenza A Viruses, Vietnam, 2020–2024

**DOI:** 10.3201/eid3210.260593

**Published:** 2026-10

**Authors:** Ngoc Anh Bui, Zoe Yi Song, Samuel M.S. Cheng, Duy Tung Dao, Lydia D.J. Chang, Gigi Y.Z. Liu, Quang Huy Nguyen, Thi Hang Dang, Quoc Hung Nguyen, Tom L.Y. Woo, Coco H.C. Chan, Tommy T.Y. Lam, Vuong N. Bui, Malik Peiris

**Affiliations:** Vietnam Institute of Animal and Veterinary Science, Hanoi, Vietnam (N.A. Bui, D.T. Dao, Quang H. Nguyen, T.H. Dang, Quoc H. Nguyen, V.N. Bui); The University of Hong Kong, Hong Kong, China (Z.Y. Song, S.M.S. Cheng, L.D.J Chang, G.Y.Z. Liu, T.L.Y. Woo, C.H.C. Chan, T.T.Y. Lam, M. Peiris).

**Keywords:** Swine Influenza A viruses, influenza, swine, viruses, reassortment, molecular epidemiology, phylogeny, zoonoses, respiratory infections, Vietnam

## Abstract

Swine influenza A viruses (IAV-S) caused the 2009 H1N1 pandemic and pose a future zoonotic and pandemic threat. Vietnam represents a critical hotspot for IAV-S emergence within East and Southeast Asia, with dense swine and human populations and intensive livestock trade. We conducted genomic surveillance of IAV-S in Vietnam during 2020–2024, extending previous surveillance from 2013–2019. We identified multiple co-circulating H1 and H3 clades, including pandemic H1N1, Eurasian avian-like, and European lineages, by conducting phylogenetic analysis of 56 IAV-S isolates (21 H1N1, 31 H1N2, and 4 H3N2). Three H1 clades persisted exclusively in Vietnam, circulating up to 12 years. Phylogeographic analysis revealed multiple independent introduction events from North America, Europe, China, Thailand, and Cambodia. We detected extensive reassortment that frequently involved pandemic H1N1 virus internal genes. We identified several lineage-specific mutations associated with mammalian adaptation. Our findings underscore the ongoing IAV-S evolution and need for sustained surveillance in Vietnam.

Swine influenza A viruses (IAV-S) contribute to zoonotic and pandemic risk because of their genetic diversity, rapid evolution, and interspecies transmission (*1*). Swine are evolutionary intermediaries, enabling reassortment between avian, human, and endemic swine influenza viruses and generating genotypes with pandemic potential (*2*). Circulation in swine might also enable the adaptation of avian virus hemagglutinins to bind to α2,6-linked sialic acids receptors found in the mammalian upper respiratory tract. This unique role was dramatically illustrated by the 2009 H1N1 pandemic, caused by a reassortant swine virus containing gene segments from classical swine, Eurasian avian-like swine, and human seasonal virus lineages (*3*).

Globally, influenza A virus subtypes H1N1, H1N2, and H3N2 are endemic in swine populations, displaying extensive genetic and antigenic diversity across different geographic regions. A phylogenetic nomenclature system for IAV-S defines 3 lineages for IAV-S H1, the classical swine lineage (1A, including the 2009 H1N1 pandemic virus [pH1N1]), the pre-2009 human seasonal lineage (1B), and the Eurasian avian-like lineage (1C). IAV-S H3N2 are defined by the introduction from human seasonal virus and circulation decade (1970, 1990, 2000, and 2010) (*4*). The evolutionary dynamics of IAV-Ss are influenced by multiple human seasonal influenza spillovers into swine, followed by sustained within-host transmission and rapid adaptation (*5*). The diversification of IAV-Ss is amplified by the intensive global swine production and international trade, which enables the movement of live swine and viruses across continents, heightening the risk for zoonotic emergence (*6*).

East and Southeast Asia is a critical hotspot for the emergence and evolution of IAV-Ss. High, dense human and swine populations with poultry production, including live-bird markets, enabled frequent reassortment and interspecies transmission in the region (*7*). Vietnam is the second largest swine producer in Asia other than China (https://www.fas.usda.gov/data/production/0113000). Longitudinal IAV-S surveillance was initiated in 2013 at the Van Phuc slaughterhouse in Hanoi, Vietnam, which sources pigs from 23 provinces across the country (*8*). Surveillance data from 2013–2019 revealed the co-circulation of diverse H1N1, H1N2, and H3N2 subtypes and demonstrated the persistence of swine-origin H1N2 and H3N2 viruses, likely introduced by the swine trade from other Asian countries and North America. The mix of the viruses was shaped by repeated reverse zoonotic introductions of human-origin pH1N1 viruses and extensive reassortment events, with H1N2 and H3N2 genotypes frequently incorporating pH1N1 internal gene segments (*9*). Strengthened surveillance and molecular characterization of those viruses in Vietnam are crucial for timely risk assessment and regional preparedness. We report findings from Van Phuc slaughterhouse surveillance during 2020–2024.

## Methods

### Sample Collection

During October 2020–April 2024, we collected nasal swab specimens monthly at Van Phuc slaughterhouse, the main centralized swine slaughterhouse in Hanoi, Vietnam. Multiple trucks transport pigs to the abattoir from farms, primarily from North Vietnam. The pigs on each truck might originate from a single farm or be consolidated from several farms within the same region. Upon arrival in the abattoir, the pigs are distributed into multiple holding pens, each assigned to a specific slaughterer. All pigs within the pens were slaughtered on the same day, but a small residual number might be carried over to the next day, potentially mixing with newly arrived pigs. Our sampling team was divided to 3–4 groups and each group collect samples randomly from pigs in >1 pens. We collected swabs and serum samples immediately after slaughter. Pigs sampled at the facility were 4–6 months of age. On the basis of the reported origin at slaughter, 64.3% of pigs originated from the Red River Delta, 21.3% from Northern midland and mountain provinces, 13.7% from the North Central Coast, 0.5% from the Central Highlands, and 0.2% from the South-East region of Vietnam. We inserted sterile dry swabs (Copan, https://www.copanusa.com) ≈10 cm into the nasal cavity and then placed the swabs into tubes containing 1 mL viral transport medium supplemented with antimicrobial drugs and bovine serum albumin (Thermo Fisher Scientific, https://www.thermofisher.com). We transported specimens to the National Institute of Veterinary Research (Hanoi, Vietnam) in an icebox and stored at 4°C upon arrival. We initiated virus isolation on the day of collection by inoculating viral transport medium onto MDCK cells (*9*).

### Virus Isolation

We cultured MDCK cells in DMEM (Thermo Fisher Scientific) with 20 mM HEPES (Thermo Fisher Scientific) and 5% fetal bovine serum (Thermo Fisher Scientific) on 24-well cell-culture plates (TPP, https://www.tpp.ch) to confluency. We then removed all culture medium, washed the cells, and replaced the medium with DMEM with 20 mM HEPES and 2 µg/mL L-1 tosylamido-2-phenylethyl chloromethyl ketone treated trypsin. After inoculation, we incubated the cultures at 37°C; we replaced the medium with fresh culture medium the next day. We monitored the cultures for cytopathic effect at 3–4 days postinoculation and harvested supernatants for hemagglutination testing. When we detected cytopathic effect or hemagglutination, we performed a second passage. We screened putative influenza A–positive cultures by using a rapid antigen detection assay (Standard Q Influenza A&B Test; SD BIOSENSOR, https://www.sdbiosensor.com). We stored confirmed virus isolates at −80°C until shipment to the University of Hong Kong (Hong Kong, China) on dry ice.

### Whole-genome Sequencing and Assembly

We extracted viral RNA from MDCK culture isolates by using the QIAamp Viral RNA Mini Kit (QIAGEN, https://www.qiagen.com), according to the manufacturer’s instructions. We synthesized complementary DNA by using SuperScript IV reverse transcriptase (Invitrogen, https://www.invitrogen.com). We performed full-length amplification of all 8 influenza A virus gene segments by using an in-house universal multisegment reverse transcription PCR primer set and the TaKaRa LA Taq Hot Start Version kit (Takara Bio, https://www.takarabio.com) (*9*). We purified amplicons by using the QIAquick PCR Purification Kit (QIAGEN). We used purified PCR products for next-generation sequencing library preparation with Illumina DNA Prep (Illumina, https://www.illumina.com), and sequenced libraries on the iSeq 100 System (Illumina). We assembled sequence reads by using the US Centers for Disease Control and Prevention MIRA pipeline (https://github.com/CDCgov/MIRA-NF).

### Phylogenetic Analysis

We sourced influenza A virus whole-genome sequences and associated metadata from the GISAID (http://www.gisaid.org) and National Center for Biotechnology Information databases (accessed February 1, 2025). To maintain computational feasibility for phylogenetic analysis, we randomly subsampled human seasonal influenza (H3N2 and H1N1) to 1% of the available data. We then curated the dataset by removing laboratory-derived strains, those of unknown subtype, and duplicate entries identified by strain name. We aligned individual gene segments separately by using MAFFT version 7.490 (*10*). We inferred maximum-likelihood phylogenies for each gene segment by using IQ-TREE version 2.2.2.6 (https://iqtree.github.io) under the general time reversible plus empirical frequencies plus free rate 4 model (*11*). We assessed branch support with 1,000 Shimodaira-Hasegawa–like approximate likelihood-ratio test replicates (*12*). We reconstructed a time-scaled phylogeny by using TreeTime version 0.11.2 (*13*). We identified clades for H1 and H3 according to the global IAV-S nomenclature system (*4*,*14*). We reconstructed ancestral states representing viral clades on the phylogeny by using PASTML version 1.9.42 with the DELTRAN (delayed transformation) algorithm (*15*). We identified reassortment events as internal nodes where the inferred clade state changed between ancestral and descendant nodes.

## Results

### Persistence of Clades in Vietnam

Phylogenetic analysis identified 3 H1 clades, 1B.2.2.1, 1B.2.9, and 1C.2.4, that were detected persistently only in Vietnam among all monitored Asia countries (Figure 1). In contrast, the Eurasian avian-like clade 1C.2.3 was first introduced into China before subsequently spreading to South Korea and Vietnam. For H3 viruses, the circulation of clades 3.2000.4 and 3.1990.4.c in Vietnam occurred contemporaneously with clade detection in China and South Korea, whereas the introduction of clade 3.1990.3 into Vietnam came after its initial establishment in Thailand. That pattern highlights Vietnam’s role as both a recipient and a source within the regional network of IAV-S transmission.

**Figure 1:**
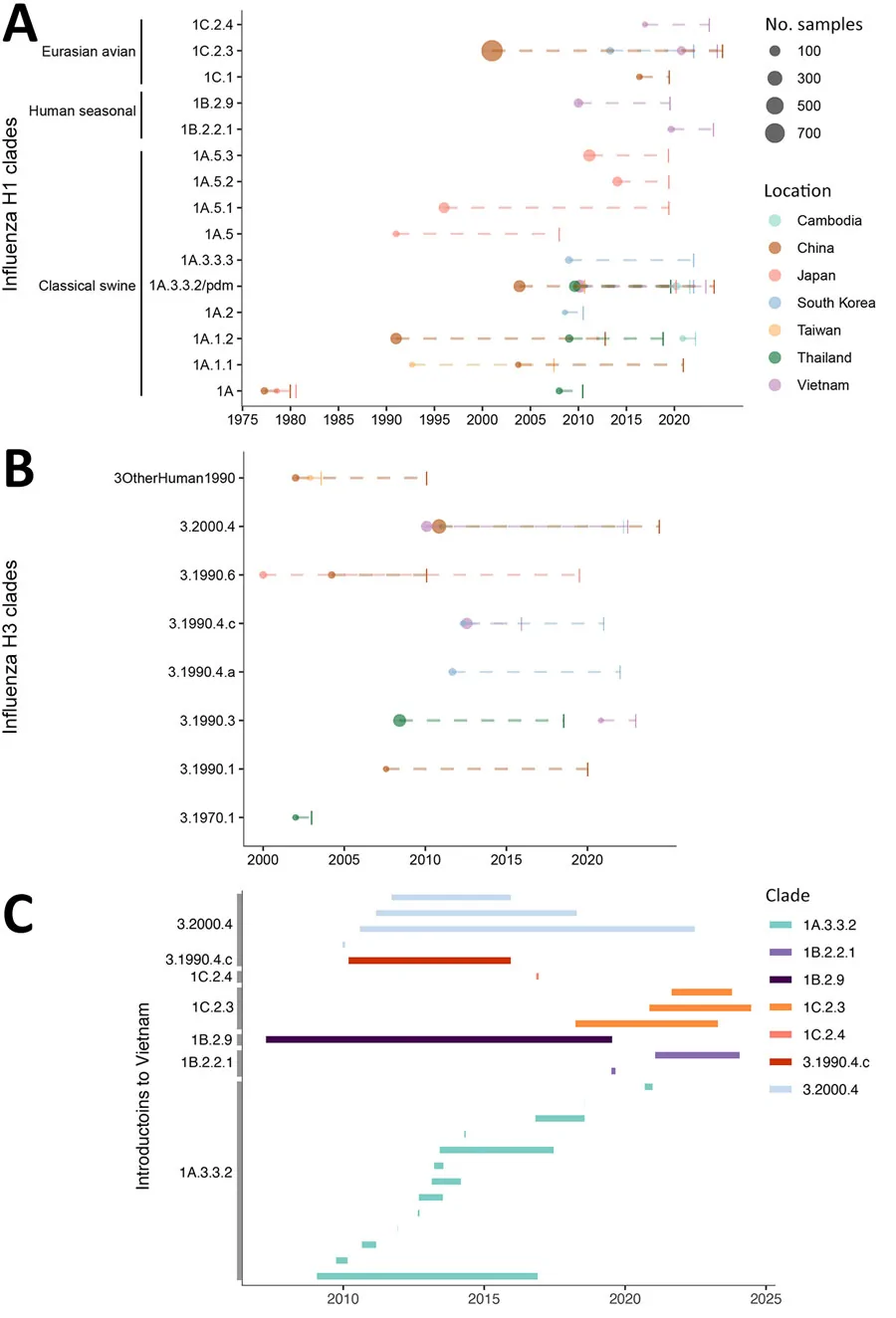
Duration of swine influenza clades circulated in Asia for H1 (A) and H3 (B) and clusters of each clade introduced into Vietnam (C) as part of study of molecular epidemiology and evolution of swine influenza A viruses, Vietnam, 2020–2024. In panels A and B, the dots indicate the sampling dates of the earliest viruses of each clade displayed, the vertical bars are the times of the latest samples, and the size of the dots represents the total number of samples of the clade in that location. In panel C, each bar starts from the time of the most recent common ancestor and the sampling time of the latest virus of each clade.

### Introduction and Reassortment of IAV-S in Vietnam

Our surveillance uncovered multiple lineages co-circulating in Vietnam during 2020–2024. We detected 21 H1N1 and 31 H1N2 isolates from clades 1A.3.3.2/pdm, 1B.2.2.1, and 1C.2.4 and 4 H3N2 isolates from clades 3.2000.4 and 3.1990.3 (Figures 2, 3).

**Figure 2:**
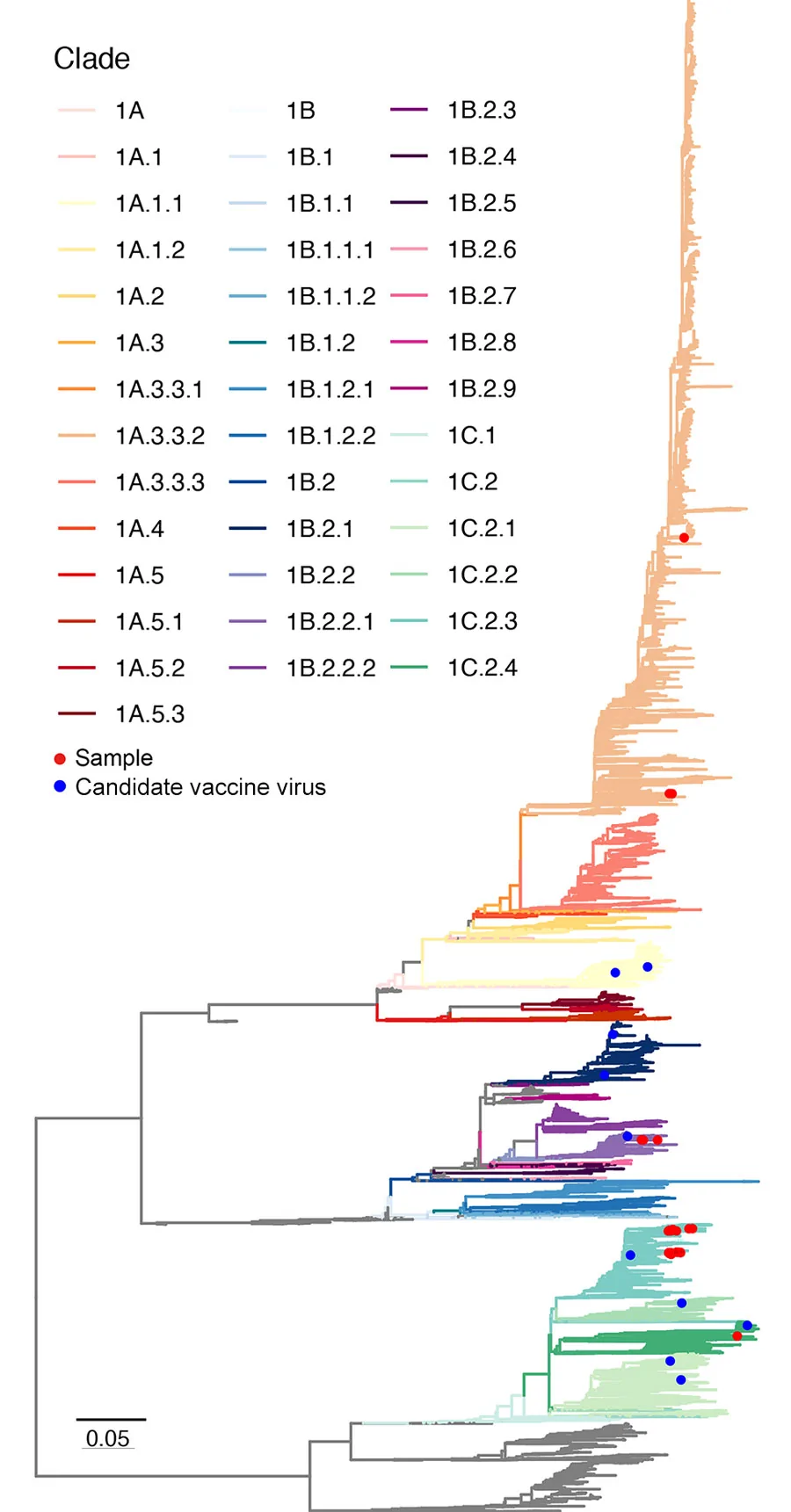
Maximum-likelihood tree of influenza A virus H1 gene from study of molecular epidemiology and evolution of swine influenza A viruses, Vietnam, 2020–2024. Branches are colored by clades. Scale bar represents the number of nucleotide substitutions per site.

**Figure 3:**
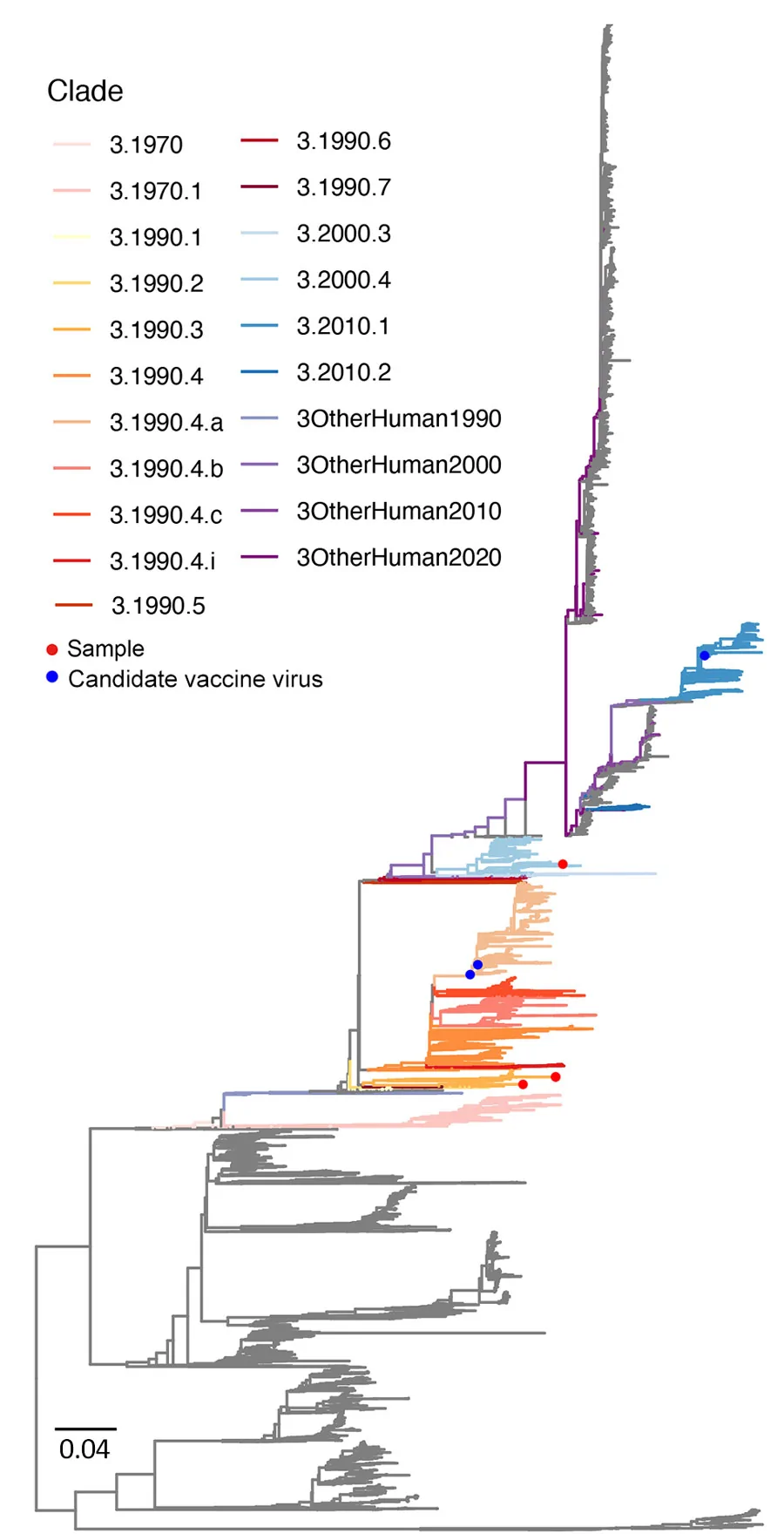
Maximum-likelihood tree of influenza A virus H3 gene from study of molecular epidemiology and evolution of swine influenza A viruses, Vietnam, 2020–2024. Branches are colored by clades. Scale bar represents the number of nucleotide substitutions per site.

Within the Eurasian avian-like lineage, clade 1C.2.3 sequences from Vietnam clustered phylogenetically with isolates from China; however, long branches and major gaps in sampling time indicated periods of unsampled diversity and cryptic local circulation. Two samples from 2021 and 2023 clustered closely with viruses from Vietnam from 2020, providing evidence for the sustained persistence of this specific viral cluster for >3 years. The 1C.2.3 viruses exchanged gene segments with co-circulating clades 1A.3.3.2/pdm, 1B.2.9, 1B.2.2.1, and 1C.2.4 (Figure 4). Clade 1C.2.4 viruses in Vietnam originated from sporadic introductions from European swine populations, having acquired internal genes from the 1A.3.3.2/pdm, 1B.2.2.1, and 3.2000.4 lineages (Figure 5).

**Figure 4:**
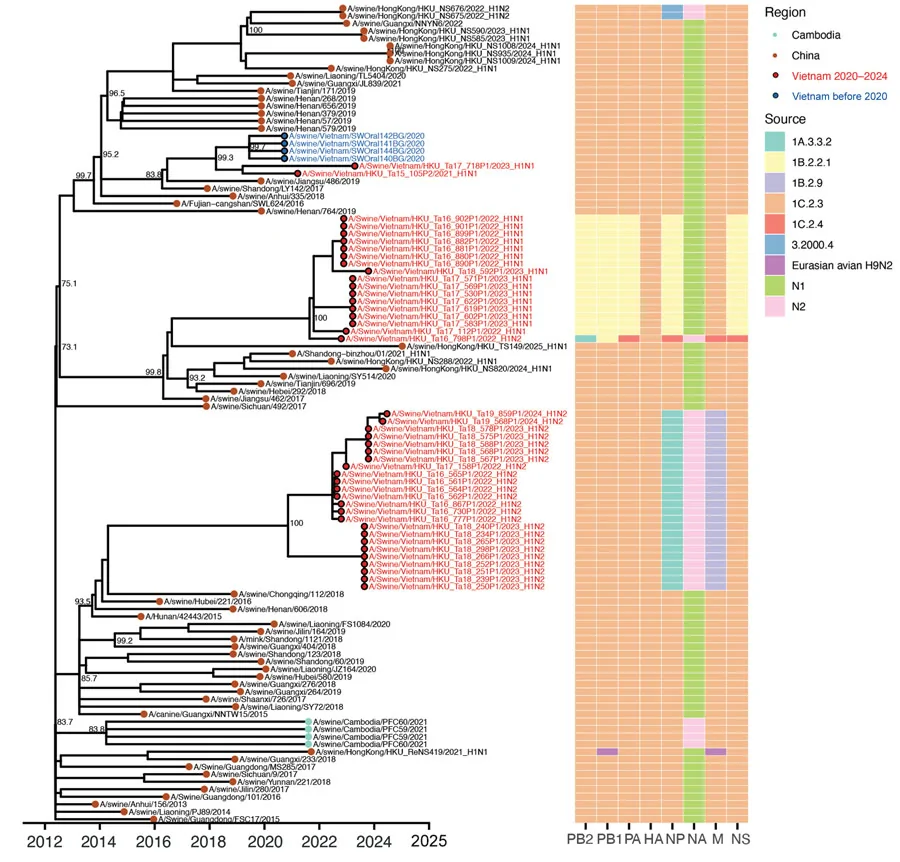
Time-scaled maximum likelihood phylogeny of swine influenza A virus hemagglutinin H1 clade 1C.2.3 from study of molecular epidemiology and evolution of swine influenza A viruses, Vietnam, 2020–2024. The heatmap shows the genetic source of all genes. Dots are colored by geographic regions. Red and blue circled dots and labels show the samples in Vietnam from this study and from previous studies. Shimodaira-Hasegawa–like approximate likelihood ratio test branch support values are labeled on nodes. The horizontal axis represents calendar time.

**Figure 5:**
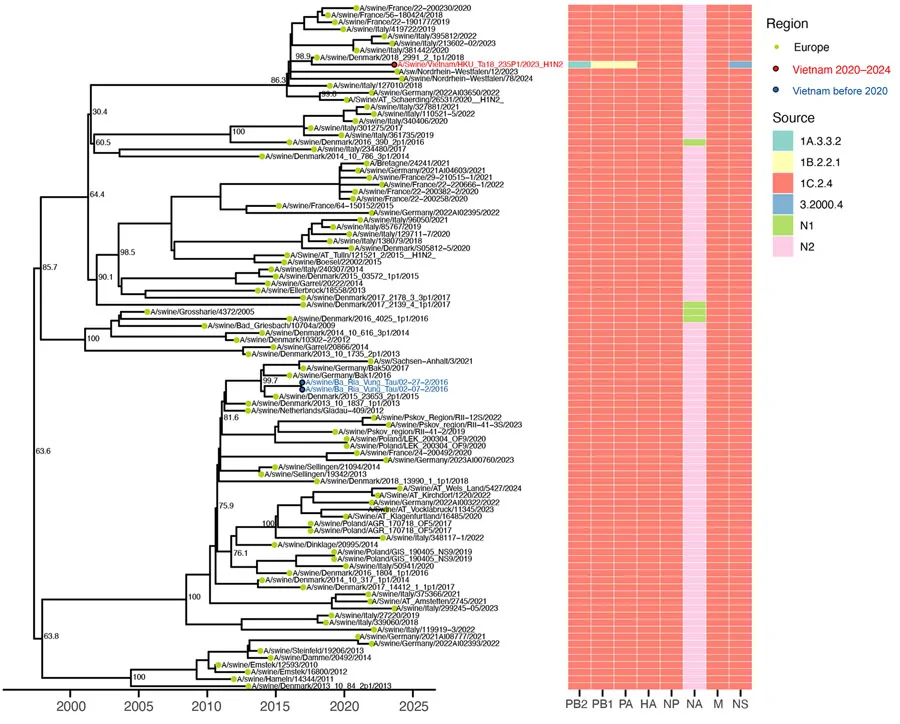
Time-scaled maximum likelihood phylogeny of swine influenza A virus hemagglutinin H1 clade 1C.2.4 from study of molecular epidemiology and evolution of swine influenza A viruses, Vietnam, 2020–2024. The heatmap shows the genetic source of all genes. Dots are colored by geographic regions. Red and blue circled dots and labels show the samples in Vietnam from this study and from previous studies. Shimodaira-Hasegawa–like approximate likelihood ratio test branch support values are labeled on nodes. The horizontal axis represents calendar time.

We detected 3 monophyletic Vietnam 1B clusters. The oldest cluster, circulating during 2010–2019, was not described in the most recent global nomenclature. We therefore refer to it as a proposed new clade, 1B.2.9, pending formal nomenclature review. The other 2 clusters fell into clade 1B.2.2.1, each representing independent introduction events from the United States. The smaller one only included Vietnam viruses from 2019. The more recent cluster exhibited continuous evolutionary progression from 2021–2024, indicating sustained local transmission after initial introduction (Figure 6). We identified no reassortment in clade 1B.2.2.1 genomes. Clade 1A.3.3.2, corresponding to the pH1N1 lineage, showed evidence of constant reverse zoonotic transmissions from humans to swine populations (Figure 7).

**Figure 6:**
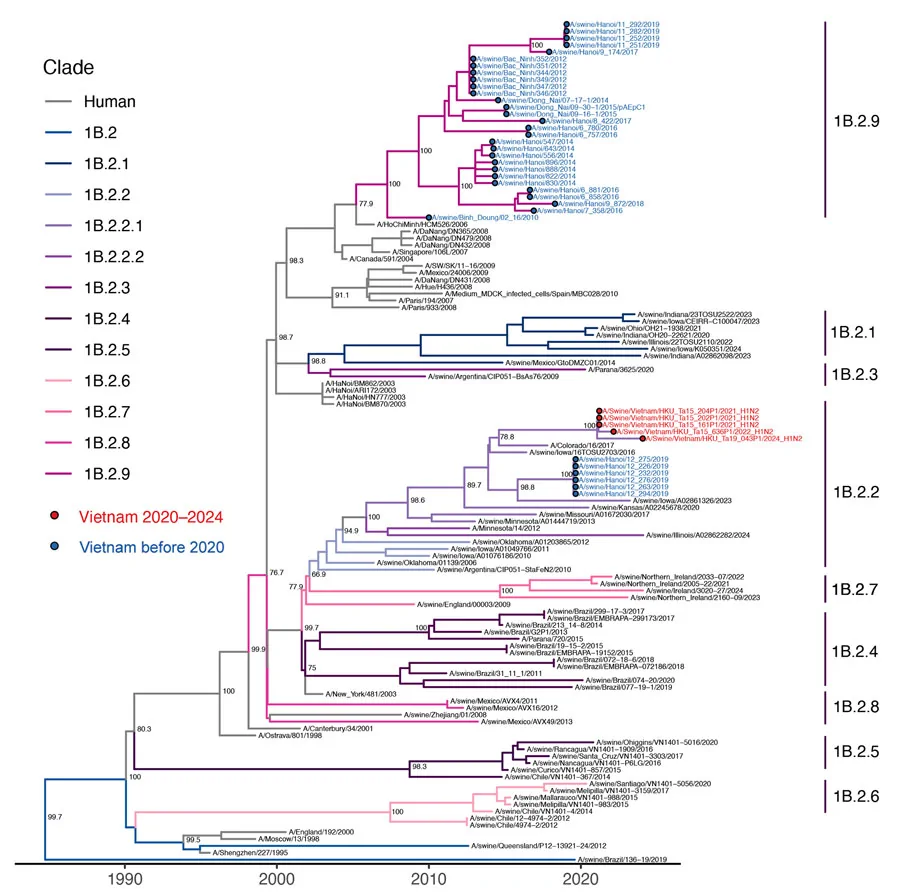
Time-scaled maximum likelihood phylogeny of swine influenza A virus hemagglutinin H1 lineage 1B from study of molecular epidemiology and evolution of swine influenza A viruses, Vietnam, 2020–2024. Branches colored by lineage 1B clades. Red and blue circled dots and labels show the samples in Vietnam from this study and from previous studies. Shimodaira-Hasegawa–like approximate likelihood ratio test branch support values are labeled on nodes. The horizontal axis represents calendar time.

**Figure 7:**
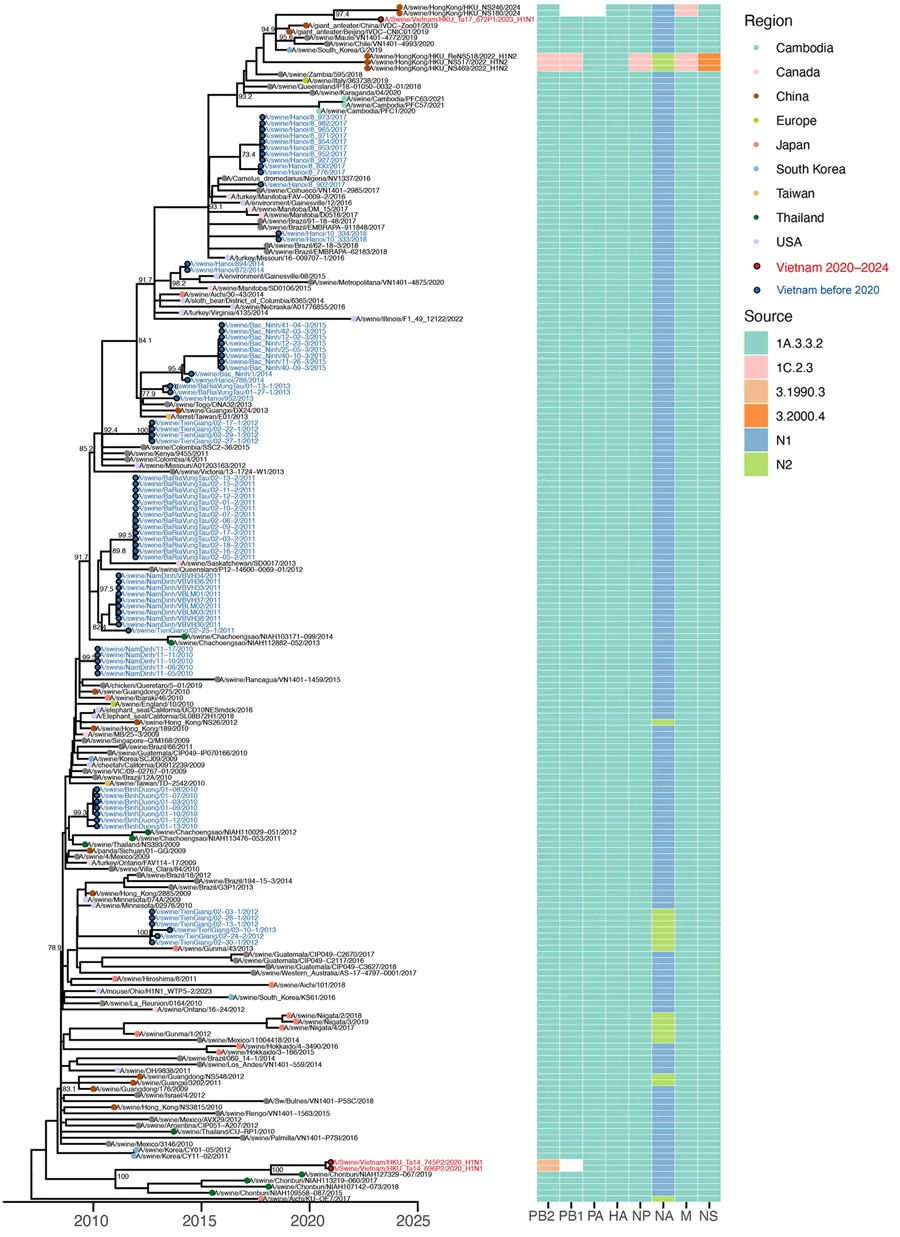
Time-scaled maximum likelihood phylogeny of swine influenza A virus hemagglutinin H1 clade 1A.3.3.2/pdm from study of molecular epidemiology and evolution of swine influenza A viruses, Vietnam, 2020–2024. Tips colored by geographic region. Red and blue circled dots and labels show the samples in Vietnam from this study and from previous studies. Shimodaira-Hasegawa–like approximate likelihood ratio test branch support values are labeled on nodes. The horizontal axis represents calendar time.

H3 clade 3.2000.4 demonstrated sustained persistence in Vietnam, with continuous detection from its initial identification in 2010–2022 (Figure 8). Phylogenetic analysis revealed that the most recent Vietnam isolates clustered with isolates from Cambodia and China, suggesting active cross-border transmission within the East and Southeast Asian region. Clade 3.1990.3 viruses detected in Vietnam clustered most closely with viruses from Thailand and had reassorted with internal genes from clades 1A.1.2 (circulating in Thailand) and 1A.3.3.2/pdm. The phylogenetic relationships were compatible with >2 introductions from Thailand or from an unsampled related source in the region, although sparse regional surveillance limits definitive source attribution (Figure 9). Clade 3.1990.4.c samples were detected during 2012–2015 but absent from recent surveillance samples, suggesting either local extinction or displacement by competing lineages.

**Figure 8:**
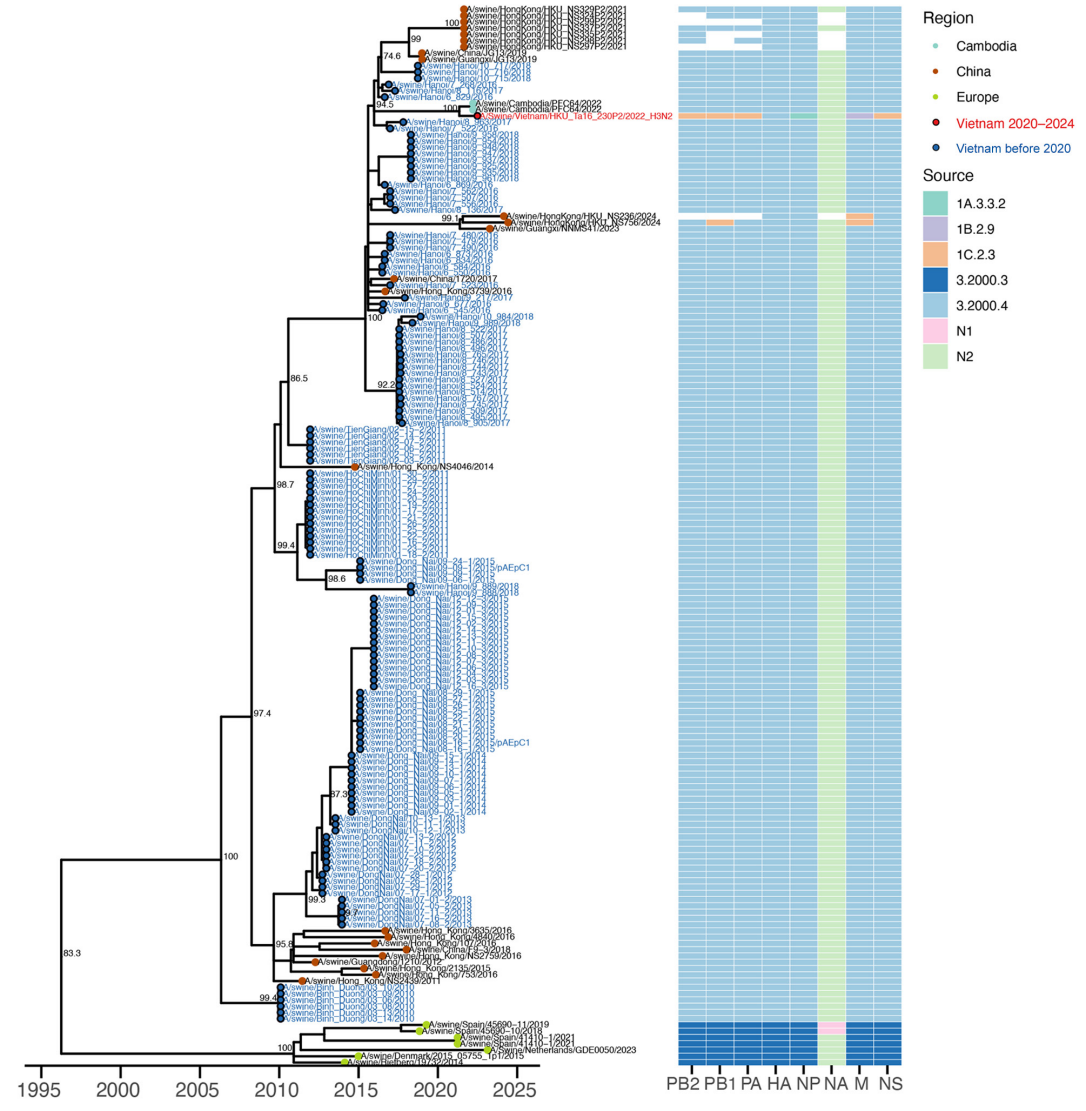
Time-scaled maximum likelihood phylogeny of swine influenza A virus hemagglutinin H3 clade 3.2000.4 from study of molecular epidemiology and evolution of swine influenza A viruses, Vietnam, 2020–2024. The heatmap shows the genetic source of all genes. Dots are colored by geographic regions. Red and blue circled dots and labels show the samples in Vietnam from this study and from previous studies. Shimodaira-Hasegawa–like approximate likelihood ratio test branch support values are labeled on nodes. The horizontal axis represents calendar time.

**Figure 9:**
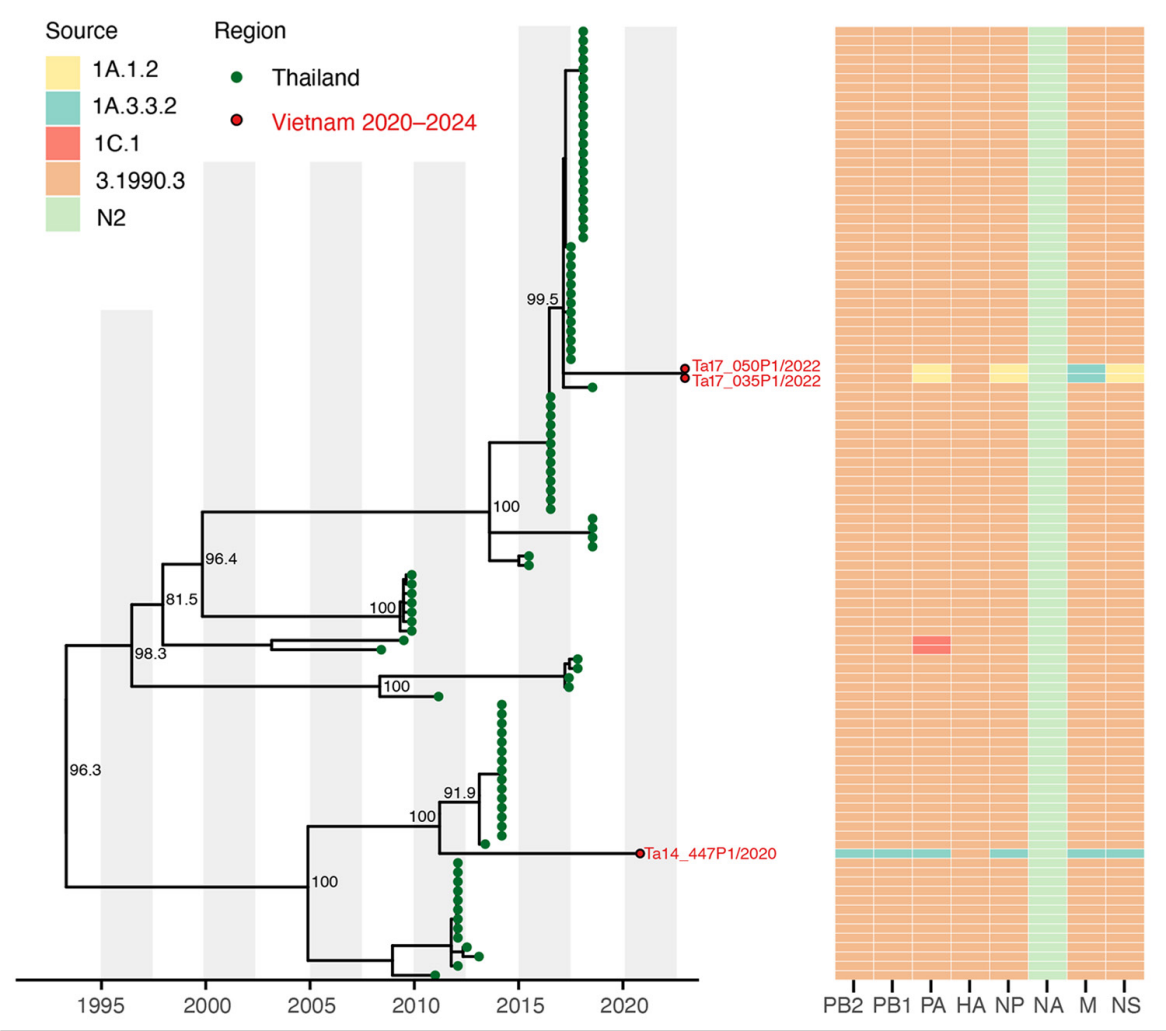
Time-scaled maximum likelihood phylogeny of swine influenza A virus hemagglutinin H3 clade 3.1990.3 from study of molecular epidemiology and evolution of swine influenza A viruses, Vietnam, 2020–2024. The heatmap shows the genetic source of all genes. Dots are colored by geographic regions. Red and blue circled dots and labels show the samples in Vietnam from this study and from previous studies. Shimodaira-Hasegawa–like approximate likelihood ratio test branch support values are labeled on nodes. The horizontal axis represents calendar time.

### Mutations Across Lineages

Analysis of mutation patterns across Vietnam IAV-S lineages revealed lineage-specific adaptive mutations involving polymerase activity, receptor binding, and virulence (Appendix Figure) (*16*,*17*). The K389R substitution in polymerase basic (PB) 2 gene, found in clade 1C.2.3, was acquired from reassortment with 1B.2.2.1. We detected the D701E substitution in 1 H1N2 isolate from 2022. That change affects the same PB2 residue as the well-characterized mammalian-adaptive substitution D701N, but it is a different amino-acid replacement, and its phenotypic effect requires experimental confirmation. K158N in the polymerase acidic gene emerged from the evolution within Vietnam IAV-S in 2023 and sustained in the swine population in 2024. We found H1 Q189E (H3 numbering Q192E) within the Sb antigenic site in a monophyletic IAV-S 1C.2.3 cluster in 2023, which we also found in swine samples from 1A.3.3.2/pdm in 2023. Nonstructural 1 gene mutations N127K and S189G emerged in clade 1C.2.3, resembling the 189G in other human-related swine samples.

## Discussion

This analysis extended IAV-S surveillance in Vietnam and revealed evolutionary and epidemiologic patterns characterizing IAV-S in Vietnam. Lineages circulating in Vietnam arose through repeated introductions from various geographic regions, including North America, Europe, and other regions of Asia, likely enabled by international swine trade. Importation of live pigs for breeding stock from Europe and North America does occur. Illegal cross-border pig imports could potentially happen, but there are no data on such movements. Most H1 lineages and H3 clade 3.1990.3 exhibited evidence of multiple independent introduction events, whereas clades 1B.2.9 and 3.2000.4 demonstrated sustained local persistence for 9–12 years after initial introduction. Frequent reassortment occurred among co-circulating lineages, generating extensive genomic diversity through gene segment exchange. We detected frequent genetic mixing between human pH1N1 and endemic swine influenza viruses in reassortant viruses. Those findings collectively demonstrate the complex evolutionary dynamics driving IAV-S diversity in Vietnam, shaped by international virus introductions, local persistence and evolution, interlineage reassortment, and reverse zoonosis from humans.

Recent surveillance highlights region-specific IAV-S dominance. In North America, IAV-S diversity is driven mainly by clades 1A.3.3.3-c3, 1B.2, and 3.1990.4.a. In Europe, swine populations show co-circulation of clades 1A.3.3.2/pdm, 1B.1, and 1C, with frequent 1C.2.4 detections. China has sustained prominent 1C.2.3 viruses with pH1N1-derived internal genes (*18*–*20*). Vietnam shares those global patterns through clades 1A.3.3.2/pdm, North America–like 1B.2.2.1, and Eurasian avian-like 1C.2.3 but differs by showing prolonged local persistence of proposed 1B.2.9 and H3 3.2000.4. Vietnam IAV-Ss clustered closely with viruses from China, South Korea, Thailand, and Cambodia, indicating frequent inter-regional transmission within East and Southeast Asia. Phylogeographic analyses have demonstrated that south central China serves as a dominant source of IAV-S dissemination to other regions in China and Southeast Asia, with strong migration pathways extending to northeast China and subsequently to Vietnam (*21*). That spatial diffusion pattern is driven by factors including pig production levels, trade networks, and pork consumption patterns.

Livestock movement can act as a catalyst for viral diversification. Pigs are often transported over long distances from farms to central slaughterhouses in Vietnam, a process that involves mixing of animals from different sources. Research from Hong Kong, which imports >90% of its swine from mainland China, has demonstrated that the transport and marketing stages of the supply chain provide a high-risk environment for IAV-S transmission. The force of infection during transport was estimated to be 229%–414% higher per unit time than within farms, enabling cross-infections and potential reassortment among viruses from different geographic origins (*22*).

Reassortments occurred among co-circulating IAV-Ss in Vietnam. pH1N1-derived internal gene segments in IAV-Ss were detected in reassortant H1N2 and H3N2 viruses, reflecting a global phenomenon documented across multiple regions. In the United States, the matrix gene from pH1N1 rapidly replaced triple-reassortant matrix genes (*23*,*24*). Similarly, in Thailand and China, reverse zoonotic transmission of pH1N1 into swine populations generated genetically distinct sublineages with predominantly pH1N1-derived internal genes (*25*–*27*). Introductions of human pH1N1 viruses into swine populations from Vietnam underscored continuous reverse zoonotic transmission at the human-animal interface. This reassortment process drives rapid IAV-S evolution and expansion of genotypic diversity.

Mutations associated with polymerase activity and mammalian adaptation were detected across multiple lineages. The K389R substitution in the PB2 gene, prominently identified in clade 1C.2.3, highly increased polymerase activity in mammalian cells through enhanced viral transcription and replication (*28*). The D701E substitution, detected exclusively in an H1N2 isolate in 2022, represents a divergent mammalian-adaptive pathway compared with the canonical D701N mutation, which is well-characterized as promoting adaptation to mammalian hosts through enhanced nuclear localization signal exposure and importin-α binding (*29*). The D701E variant suggests alternative adaptive strategies emerging within Vietnam swine populations. Q189E (H3 numbering Q192E) was detected in clade 1C.2.3 and 1A.3.3.2/pdm samples in 2023 in Vietnam. The Q189E located in the Sb antigenic epitope represents antigenic drift driven by immune selection pressures. This mutation parallels substitutions seen in the H1N1 clade 6B.1A.5a and its descendants that were prevalent in the Northern Hemisphere since the 2019–2020 influenza season (*30*). In Vietnam, the World Health Organization (WHO) reported a laboratory-confirmed human infection with swine-origin influenza A(H1N1) variant virus in Son La province in 2024; no additional respiratory illness was reported among contacts, and WHO assessed the risk to the general population as low (*31*). Subsequent WHO genetic and antigenic characterization classified this virus as H1 clade 1C.2.3 and described it as closely related to swine viruses circulating in the region, with good reactivity to postinfection ferret antiserum raised against the clade 1C.2.3 candidate vaccine virus A/Hunan/42443/2015 (*32*). Clade 1C.2.3 was detected in Vietnam swine in our surveillance during 2020–2024, but public summaries of the human case do not report whether the human virus contained any specific substitutions. Human variant infections demonstrated that some lineages detected in swine can cross the species barrier, although current evidence supports sporadic zoonotic transmission rather than sustained human-to-human spread.

The first limitation of this study is that molecular detection methods might yield higher detection rates of virus, but virus isolation has the advantage of providing viruses for further phenotypic characterization. Second, our study does not provide data on swine viruses that circulate in the southern part of Vietnam. Finally, because swine are transported long distances by truck in closely confined spaces, there is opportunity for cross-infection during transport, increasing the true IAV-S infection rates in swine, compared with what might be observed in farms.

The persistent circulation and repeated introductions of multiple IAV-S lineages in Vietnam underscore the region’s risk for novel zoonotic influenza emergence. IAV-Ss circulating in Vietnam showed close phylogenetic relationships with isolates from other countries in Asia, although temporal and geographic surveillance gaps limit a complete characterization of regional transmission networks and the capacity for early detection and effective intervention.

Appendix Additional information about molecular epidemiology and evolution of swine influenza A viruses, Vietnam, 2020–2024.
