# Supplementary material for: Molecular Epidemiology and Evolution of Swine Influenza A Viruses, Vietnam, 2020–2024
Source: Emerg Infect Dis. 2026 Oct;32(10):1609–20. doi: 10.3201/eid3210.260593 (PMC13632897; doi:10.3201/eid3210.260593)
Supplement: Supplementary file 1 [file 26-0593-Techapp-s1.pdf]

Article DOI: https://doi.org/10.3201/eid3210.260593

EID cannot ensure accessibility for supplementary materials supplied by authors. Readers who have difficulty accessing supplementary content should contact the authors for assistance.

Molecular Epidemiology and Evolution of Swine Influenza A Viruses, Vietnam, 2020–2024

Appendix

Appendix Table. GISAID accession IDs for sequences generated in this study.

Virus Name	Accession ID
A/Swine/Vietnam/HKU_Ta14_447/2020	20410088
A/Swine/Vietnam/HKU_Ta14_696/2020	20410089
A/Swine/Vietnam/HKU_Ta14_745/2020	20410090
A/Swine/Vietnam/HKU_Ta15_105/2021	20410091
A/Swine/Vietnam/HKU_Ta15_161/2021	20410092
A/Swine/Vietnam/HKU_Ta15_202/2021	20410093
A/Swine/Vietnam/HKU_Ta15_204/2021	20410094
A/Swine/Vietnam/HKU_Ta15_636/2022	20410095
A/Swine/Vietnam/HKU_Ta16_230/2022	20410096
A/Swine/Vietnam/HKU_Ta16_517/2022	20410098
A/Swine/Vietnam/HKU_Ta16_518/2022	20410099
A/Swine/Vietnam/HKU_Ta16_561/2022	20410100
A/Swine/Vietnam/HKU_Ta16_562/2022	20410101
A/Swine/Vietnam/HKU_Ta16_563/2022	20410102
A/Swine/Vietnam/HKU_Ta16_564/2022	20410103
A/Swine/Vietnam/HKU_Ta16_565/2022	20410104
A/Swine/Vietnam/HKU_Ta16_567/2022	20410105
A/Swine/Vietnam/HKU_Ta16_730/2022	20410106
A/Swine/Vietnam/HKU_Ta16_774/2022	20410107
A/Swine/Vietnam/HKU_Ta16_777/2022	20410108
A/Swine/Vietnam/HKU_Ta16_798/2022	20410109
A/Swine/Vietnam/HKU_Ta16_867/2022	20410110
A/Swine/Vietnam/HKU_Ta16_880/2022	20410111
A/Swine/Vietnam/HKU_Ta16_881/2022	20410157
A/Swine/Vietnam/HKU_Ta16_882/2022	20410114
A/Swine/Vietnam/HKU_Ta16_890/2022	20410115
A/Swine/Vietnam/HKU_Ta16_899/2022	20410116
A/Swine/Vietnam/HKU_Ta16_901/2022	20410117
A/Swine/Vietnam/HKU_Ta16_902/2022	20410118
A/Swine/Vietnam/HKU_Ta17_035/2022	20410119
A/Swine/Vietnam/HKU_Ta17_050/2022	20410120
A/Swine/Vietnam/HKU_Ta17_112/2022	20410121
A/Swine/Vietnam/HKU_Ta17_158/2022	20410122
A/Swine/Vietnam/HKU_Ta17_489/2023	20410123
A/Swine/Vietnam/HKU_Ta17_530/2023	20410124
A/Swine/Vietnam/HKU_Ta17_567/2023	20410125
A/Swine/Vietnam/HKU_Ta17_568/2023	20410126
A/Swine/Vietnam/HKU_Ta17_569/2023	20410128
A/Swine/Vietnam/HKU_Ta17_571/2023	20410129
A/Swine/Vietnam/HKU_Ta17_583/2023	20410130
A/Swine/Vietnam/HKU_Ta17_602/2023	20410131
A/Swine/Vietnam/HKU_Ta17_610/2023	20410132
A/Swine/Vietnam/HKU_Ta17_619/2023	20410133
A/Swine/Vietnam/HKU_Ta17_622/2023	20410134
A/Swine/Vietnam/HKU_Ta17_672/2023	20410135
A/Swine/Vietnam/HKU_Ta17_718/2023	20410136
A/Swine/Vietnam/HKU_Ta18_234/2023	20410137

Virus Name	Accession ID
A/Swine/Vietnam/HKU_Ta18_235/2023	20410138
A/Swine/Vietnam/HKU_Ta18_239/2023	20410139
A/Swine/Vietnam/HKU_Ta18_240/2023	20410140
A/Swine/Vietnam/HKU_Ta18_250/2023	20410141
A/Swine/Vietnam/HKU_Ta18_251/2023	20410142
A/Swine/Vietnam/HKU_Ta18_252/2023	20410144
A/Swine/Vietnam/HKU_Ta18_265/2023	20410145
A/Swine/Vietnam/HKU_Ta18_266/2023	20410146
A/Swine/Vietnam/HKU_Ta18_298/2023	20410147
A/Swine/Vietnam/HKU_Ta18_567/2023	20410148
A/Swine/Vietnam/HKU_Ta18_568/2023	20410149
A/Swine/Vietnam/HKU_Ta18_575/2023	20410150
A/Swine/Vietnam/HKU_Ta18_578/2023	20410151
A/Swine/Vietnam/HKU_Ta18_588/2023	20410152
A/Swine/Vietnam/HKU_Ta18_592/2023	20410153
A/Swine/Vietnam/HKU_Ta19_043/2024	20410154
A/Swine/Vietnam/HKU_Ta19_568/2024	20410155
A/Swine/Vietnam/HKU_Ta19_859/2024	20410156

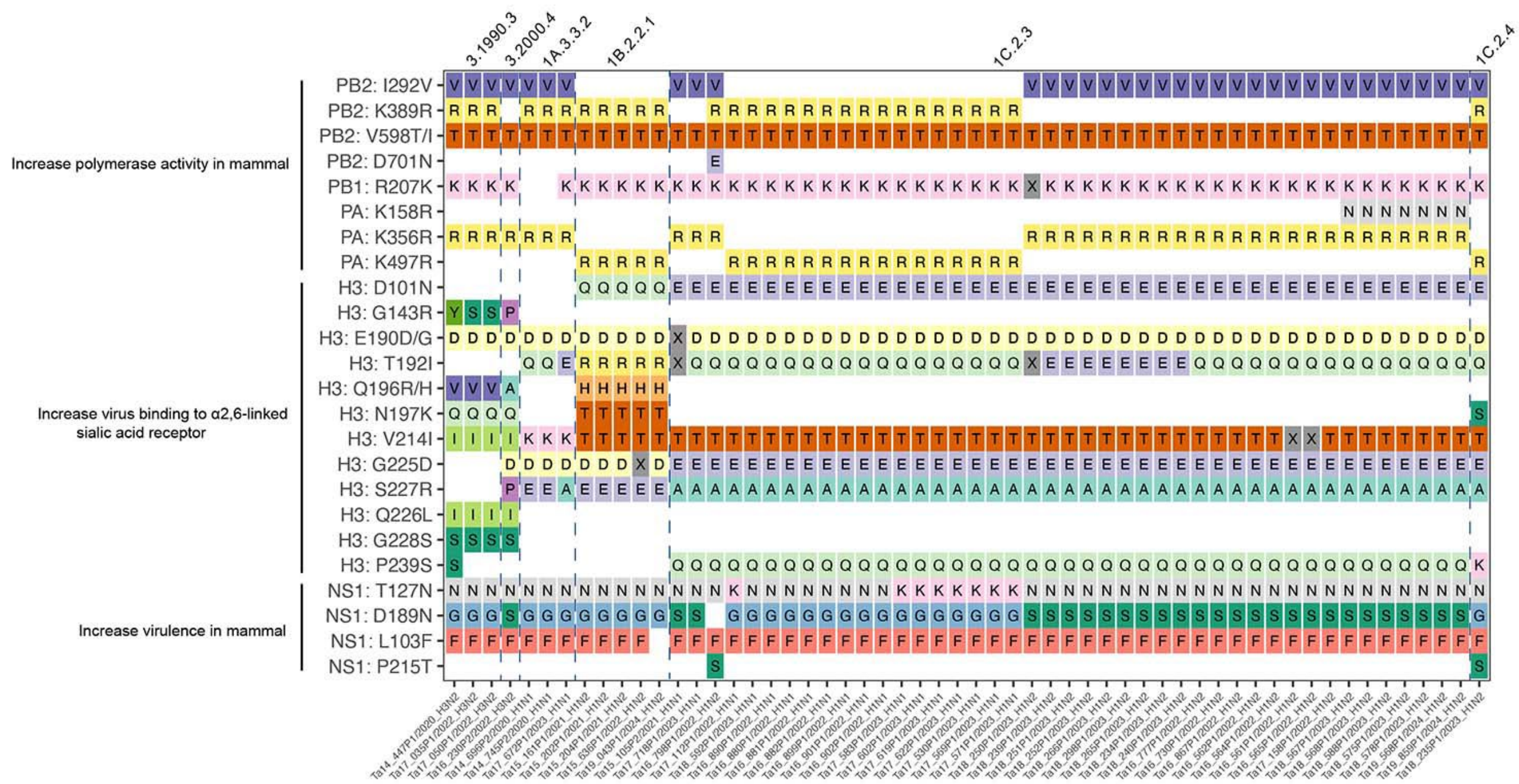

Appendix Figure. Mutations in sampled genomes that change polymerase activity, receptor binding and virulence. Blank sites represent the same amino acid as the reference.
